# Antiaging effect of a Jianpi-yangwei formula in *Caenorhabditis elegans*

**DOI:** 10.1186/s12906-019-2704-4

**Published:** 2019-11-15

**Authors:** Liling Zeng, Zhimin Yang, Tianchan Yun, Shaoyi Fan, Zhong Pei, Ziwen Chen, Chen Sun, Fuping Xu

**Affiliations:** 1 The Second Clinical College of Guangzhou University of Chinese Medicine, Guangzhou, China; 2 The Second Affiliated Hospital of Guangzhou University of Chinese Medicine, 111 Da De Rd., Yuexiu District, Guangzhou, Guangdong Province People’s Republic of China 510120; 3 Department of Neurology, National Key Clinical Department and Key Discipline of Neurology, the First Affiliated Hospital, Sun Yat-sen University, Guangzhou, China

**Keywords:** Jianpi-yangwei formula, Traditional Chinese medicine, *Caenorhabditis elegans*, Aging

## Abstract

**Background:**

Jianpi-yangwei (JPYW), a traditional Chinese medicine (TCM), helps to nourish the stomach and spleen and is primarily used to treat functional declines related to aging. This study aimed to explore the antiaging effects and mechanism of JPYW by employing a *Caenorhabditis elegans* model.

**Methods:**

Wild-type *C. elegans* N2 worms were cultured in growth medium with or without JPYW, and lifespan analysis, oxidative and heat stress resistance assays, and other aging-related assays were performed. The effects of JPYW on the levels of superoxide dismutase (SOD) and the expression of specific genes were examined to explore the underlying mechanism of JPYW.

**Results:**

Compared to control worms, JPYW-treated wild-type worms showed increased survival times under both normal and stress conditions (*P* < 0.05). JPYW-treated worms also exhibited enhanced reproduction, movement and growth and decreased intestinal lipofuscin accumulation compared to controls (*P* < 0.05). Furthermore, increased activity of SOD, downregulated expression levels of the proaging gene *clk-2* and upregulated expression levels of the antiaging genes *daf-16*, *skn-1*, and *sir-2.1* were observed in the JPYW group compared to the control group.

**Conclusion:**

Our findings suggest that JPYW extends the lifespan of *C. elegans* and exerts antiaging effects by increasing the activity of an antioxidant enzyme (SOD) and by regulating the expression of aging-related genes. This study not only indicates that this Chinese compound exerts antiaging effects by activating and repressing target genes but also provides a proven methodology for studying the biological mechanisms of TCMs.

## Background

Aging is believed to be an inevitable physiological process that occurs in all living organisms [1] and has been a concern since ancient times. Some researchers have suggested that the aging process is affected by environmental [2, 3], nutritional [4], and genetic factors [5] and have attempted to explore the mechanisms of aging. In addition, in modern times, an increasing number of aging-related diseases, such as cancer, cardiovascular disease, chronic degenerative diseases and other aging-related dysfunctions, have threatened human health [6, 7]. Even though increasing evidence has demonstrated that pharmacological intervention may delay the senescence process [8, 9], a definitely effective antiaging treatment has not yet been found since the mechanisms of aging are complicated.

In contrast to mainstream modern medicine, traditional Chinese medicine (TCM) aims to interfere with the aging process as early as possible, thus preventing and delaying the occurrence and development of aging-related diseases, and has begun to draw increasing research interest [10–12]. TCM has been used as a complementary medicine for 5000 years and has garnered much attention as a result of its high medical efficacy and its preventative functions [10, 11, 13]. In recent years, many studies have suggested that lots of TCMs exhibit an array of antiaging effects [12, 14, 15]. According to TCM theory, Jianpi-yangwei (JPYW) therapy is one of the main treatment modalities for aging and has been clinically demonstrated to be effective [16–20]; however, further research on the nature of JPYW is necessary due to the complexity of its composition. JPYW is a TCM formula that is mainly composed of 8 ingredients: *Panax ginseng C. A. Mey*, *Radix Paeoniae Alba*, *Codonopsis Radix*, *Poria cocos*, *Rhizoma Atractylodis Macrocephalae*, *Crataegus pinnatifida*, *Pericarpium Citri Reticulatae*, and *Cinnamomum cassia Presl*. In TCM theory, JPYW is based on the Sijunzi decoction, which is a classic Chinese medicine that has been demonstrated to be beneficial for the spleen and stomach as a result of its antiaging effects [19, 21]. In a previous study, a JPYW capsule was proven to have therapeutic effects on gastric precancerous lesions and cancer-related fatigue [22]. In the present study, we found that JPYW exhibited a spleen-fortifying and stomach-nourishing effect that helped to replenish energy and recover functions that were declining as a result of aging. Moreover, we drew our conclusions from ten years of clinical experience showing that JPYW has strong antiaging effects. Notably, previous studies suggested that *Caenorhabditis elegans* was a comparatively ideal model for aging research [23, 24].

This study aimed to explore the antiaging effects and the mechanism of JPYW in wild-type *C. elegans* N2 worms (Bristol). Lifespan assays, stress resistance assays and other aging-related factors and properties were assessed to evaluate antiaging effects. The activity of superoxide dismutase (SOD) and the expression levels of aging-related genes were assessed to illustrate the potential mechanisms.

## Methods

### Preparation of JPYW

JPYW mainly consists of 8 crude herbs: *P. ginseng C. A. Mey*, *Radix Paeoniae Alba*, *Codonopsis Radix*, *P. cocos*, *Rhizoma Atractylodis Macrocephalae*, *C. pinnatifida*, *Pericarpium Citri Reticulatae*, and *C. cassia Presl*. For this study, we used a mixture of water extracts of the crude herbs. The water extracts were provided by *Kangmei Pharmaceutical Co.* (Guangzhou, China), were produced according to the rigid specifications of the *Pharmacopeia of the People’s Republic of China* and were approved by the China Food and Drug Administration (CFDA). In accordance with TCM research conventions, all concentrations reported in this study refer to the concentrations of the crude herbs. The JPYW used in the study was dissolved in 1% dimethylsulfoxide (DMSO).

### *C. elegans*: strains and maintenance

The wild-type *C. elegans* N2 worms (*Bristol*) and *E. coli* OP50 were provided by the Caenorhabditis Genetics Center (CGC) (*Minneapolis*, MN, USA). The *C. elegans* strains were cultured at 20 *°C* on solid nematode growth medium (NGM) plates seeded with *E. coli* OP50. The wild-type *C. elegans* N2 worms (*Bristol*) were aged and were considered adults at 7 days.

### Lifespan analysis

A bleaching technique was used to synchronize the worm population in this study. The age-synchronized N2 nematodes were transferred to NGM plates containing 150 μg/ml JPYW or a vehicle control (1% DMSO). *E. coli* OP50 was added to the medium. Two NGM plates containing 25 worms each were used, and the worms were transferred to new NGM plates every day for the first 7 days so that the new eggs did not have a disruptive effect. Then, the survival rate was assessed every other day until the worms died. The survival fraction was calculated by recording the number of surviving worms. We considered the nematodes to be dead when there was no respond after touching them with a platinum loop (failed to exhibit touch-provoked movement). At least three independent trials of the lifespan assay were performed.

### Assessment of stress resistance

Age-synchronized N2 worms were bred on NGM plates with or without 150 μg/ml JPYW. For a heat tolerance assay, day 4 adult worms (on the 4th day after the worms reached adulthood, *n* = 50) were transferred to fresh plates containing 150 μg/ml JPYW or a vehicle control and then incubated at 37 *°C*. Survival was recorded every hour until all worms had died. The tolerance to oxidative stress was measured as reported previously [25]. Briefly, day 4 adult worms (*n* = 50) were placed on plates with various concentrations of hydrogen peroxide (from 0 mM to 1 mM, intervals of 0.2 mM) as well as 150 μg/ml JPYW or a vehicle control, and then the survival was recorded after 15 h. Each test was repeated at least twice.

### Measurement of SOD activity

To measure SOD activity, wild-type worms (n = 50) were collected from plates with M9 buffer on the 5th day of adulthood (day 5 after the worms reached adulthood) and washed 3 times. Then, the collected worms were resuspended in homogenization buffer (10 mM tris(hydroxymethyl)aminomethane hydrochloride(Tris-HCl), 150 mM NaCl, and 0.1 mM ethylenedinitrilotetraacetic acid (EDTA), pH 7.5) and homogenized through ultrasonication on ice. A total of 0.5 mg protein from every group was used to measure SOD activity. The SOD activity was spectrophotometrically analyzed on the basis of the decolorization of formazan. A Total Superoxide Dismutase (T-SOD) Assay Kit (hydroxylamine method) and a Total Protein Assay Kit (standard: bicinchoninic acid (BCA) method) were purchased from *Nanjing Jiancheng Bioengineering Institute* (Nanjing, China) and were used to determine the SOD activity and protein concentration, respectively. The procedures were performed in strict accordance with the manufacturers’ protocols.

### Measurement of aging-related factors

For a pharyngeal pumping assay, age-synchronized N2 worms (*n* = 10) were treated with 150 μg/ml JPYW or vehicle until the 4th day after the worms reached adulthood, and then their pharynx contractions were counted under an inverted microscope for 10 s in the fresh plates.

For reproduction assay, worms (*n* = 5) were cultured from eggs. Worms were individually moved to a fresh plate every day once they became adults. The progeny were counted at the L2 or L3 stage.

For a growth alteration assay, on the 4th day of adulthood, worms were photographed and their body length was analyzed by using Nikon software (*Nikon*, Japan).

For a body movement assay, age-synchronized N2 worms (*n* = 10) were bred on NGM plates with or without 150 μg/ml JPYW. On the 7th day of adulthood, their body movements expressed as the travel distance were recorded under an inverted microscope for 20 s in fresh plates, and were analyzed by using Nikon software.

The fluorescence intensity of lipofuscin and autofluorescence were assessed in the worms on the 10th day of adulthood, and were quantified using ImageJ to determine the average pixel intensity. All tests were repeated more than 2 times.

### Quantitative analysis of aging-related genes in *C. elegans*

Age-synchronized N2 worms were treated with 150 μg/ml JPYW or vehicle at 20 *°C* until the 4th day after the worms reached adulthood. Total RNA was extracted from approximately 600 worms per group with TRIzol (*TaKaRa,* Beijing, China). For RNA extraction and quantitative real-time polymerase chain reaction (qRT-PCR), more detailed steps have been described in the previous study [26]. Briefly, the collected worms were moved to 1.5-ml RNase-free microfuge tubes to extract RNA and the RNA concentration was quantified using a NanoDrop spectrophotometer. Complementary DNA (cDNA) was synthesized by reverse transcription using a PrimeScript RT Reagent Kit with gDNA Eraser (Perfect Real Time; *TaKaRa*, Beijing, China) according to the manufacturer’s protocol. Quantitative real-time polymerase chain reaction (qRT-PCR) was performed using TB Green Premix Ex Taq II (Tli RNase H Plus; *TaKaRa*, Beijing, China) with SuperReal PreMix Plus (SYBR Green; *TaKaRa*, Beijing, China). The primers were as follows: act-1, 5-TCCCTCTCCACCTTCCAACA-3 (forward) and 5-GCACTTGCGGTGAACGATG-3 (reverse); skn-1, 5-CCAGTGACAACGAGCTTCCA-3 (forward) and 5-GTGACGATCCGTGCGTCTTT (reverse); clk-2, 5-ACTCCGATCTACTCGCCTCA-3 (forward) and 5-GATGCAGGCAGTCCGTAGTT-3 (reverse); sod-3, 5′-CCAACCAGCGCTGAAATTCAATGG-3′ (forward) and 5′- GGAACCGAAGTCGCGCTTAATAGT-3′ (reverse); daf-16, 5′- CCAGACGGAAGGCTTAAACT-3′ (forward) and 5′-ATTCGCATGAAACGAGAATG-3′ (reverse). The cDNA was produced using random 6-mers and oligo (dT) primers. qRT-PCR was performed using SYBR green as the detection method. The comparative 2^−ΔΔCT^ method was used to assess the expression levels of each mRNA relative to those of *act-1*. The test was performed in triplicate.

### Statistical analyses

All the datas in the study were analyzed by using GraphPad Prism 6.0. Kaplan-Meier survival analysis and log-rank test were conducted for the lifespan assay. Student’s t-test was used for comparing two datasets. For all the datas, the mean and standard error of the mean (SEM) were analyzed. *P* values < 0.05 were considered to indicate significance.

## Results

### Effects of JPYW on lifespan extension and stress resistance

**Fig. 1:**
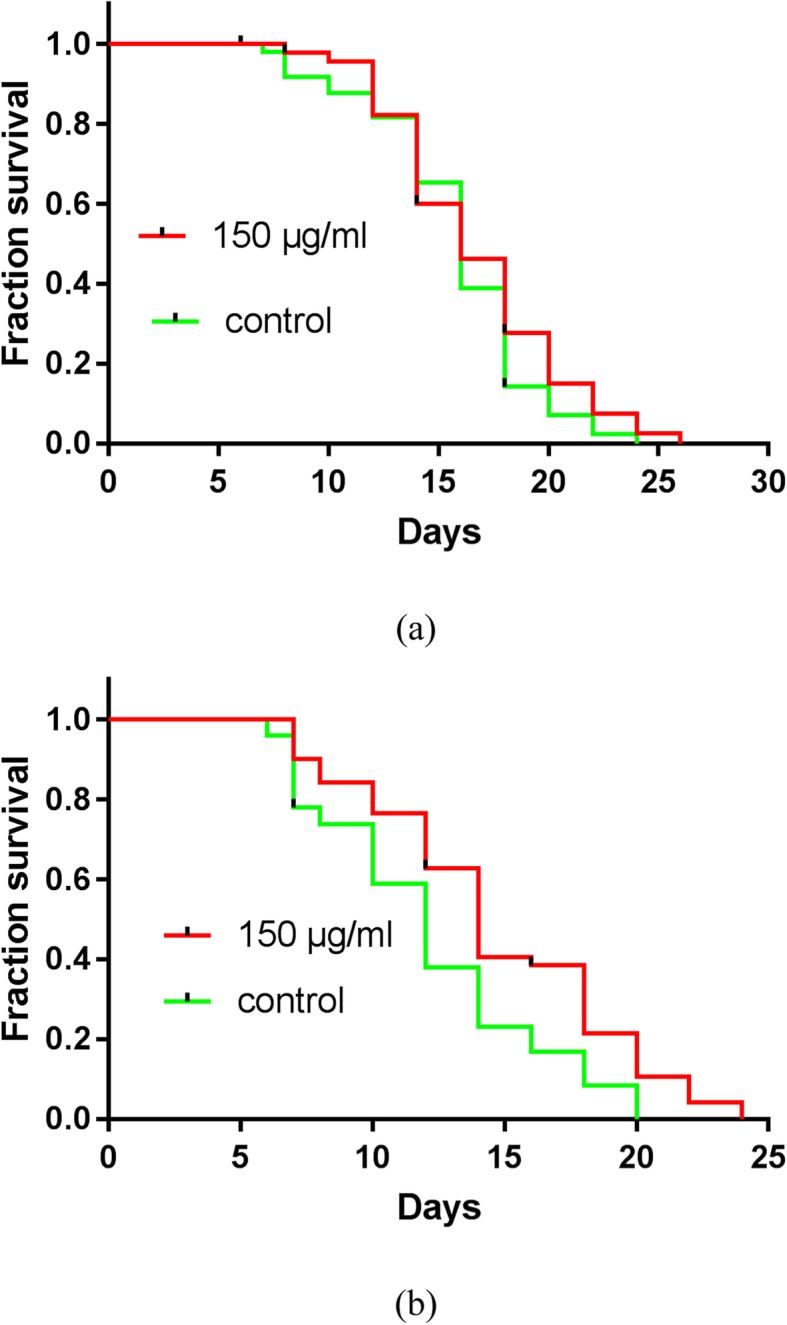
Effect of JPYW on the lifespan of *C. elegans* N2 worms under normal conditions. **a** The worms were treated with JPYW beginning at the larval stage. The curves show the percentages of surviving worms on different days after treatment with a vehicle control (1% DMSO) or 150 μg/ml JPYW. JPYW did not significantly prolong the lifespan of wild-type worms, but it caused a positive trend in the number of surviving aged worms (*n* = 50). **b** The aged worms were exposed to JPYW beginning on the 7th day of adulthood. The curves show the percentages of surviving worms on different days after treatment with a vehicle control (1% DMSO) or 150 μg/ml JPYW. JPYW significantly prolonged the lifespan of aged wild-type worms; n = 50–51, *P* < 0.05

**Fig. 2:**
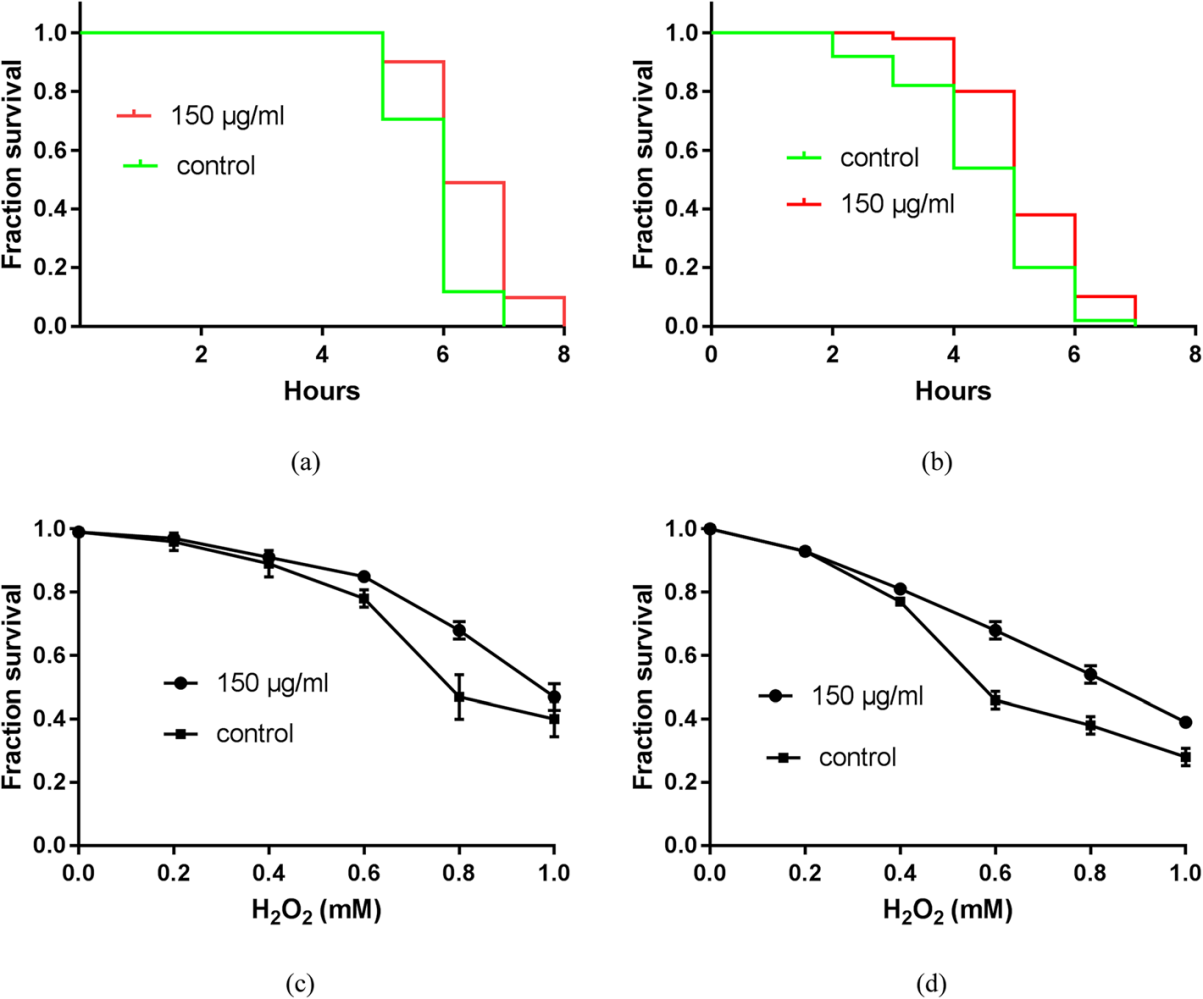
The effect of JPYW on stress resistance in *C. elegans* N2 worms. **a** Heat stress resistance in wild-type larvae. Wild-type worms that were incubated at a constant temperature (37 *°C*) were pretreated with 150 μg/ml JPYW or vehicle control (1% DMSO). Survival was assessed every hour after heat stress treatment. JPYW significantly prolonged the lifespan of wild-type worms under heat stress compared to the vehicle control (n = 50–55, *P* < 0.05). **b** Heat stress resistance in aged *C. elegans* N2 worms. Aged worms that were incubated at a constant temperature (37 *°C*) were pretreated with 150 μg/ml JPYW or vehicle control (1% DMSO). Survival was assessed every hour after heat stress treatment. JPYW treatment significantly prolonged the lifespan of aged wild-type worms under heat stress compared to the control treatment (n = 50–55, *P* < 0.05). **c** Oxidative stress resistance in *C. elegans* N2 worms. Wild-type worms were pretreated with 150 μg/ml JPYW or vehicle control (1% DMSO) and were exposed to various concentrations of hydrogen peroxide (0, 0.2, 0.4, 0.6, 0.8, and 1 mM). Survival was assessed after 15 h of each treatment. **d** Oxidative stress resistance in aged *C. elegans* N2 worms. Aged worms were pretreated with 150 μg/ml JPYW or vehicle control (1% DMSO) and were exposed to various concentrations of hydrogen peroxide (0, 0.2, 0.4, 0.6, 0.8, and 1 mM). Survival was assessed after 15 h of each treatment

To determine the lifespan-extending properties of JPYW, lifespan assays were performed using wild-type worms with or without 150 μg/ml JPYW treatment. We found significantly more worms in the old-age phase among the JPYW-treated worms than among the controls (Fig. 1a). Therefore, we hypothesized that JPYW may affect the lifespan of worms without affecting worm development. We subsequently used aged wild-type worms (7-day-old adult worms) as the experimental models for the lifespan assay. Interestingly, after 7 days of treatment, there was a significant difference between the JPYW group and the control group for every day; in addition, compared to control worms, JPYW-treated worms displayed significant increases in lifespan (11.86 ± 4.24 vs. 14.49 ± 4.78 days, *P* < 0.05) (Fig. 1b). To evaluate stress resistance, we performed heat stress assays and oxidative stress assays using wild-type worms with or without 150 μg/ml JPYW treatment. As shown in Fig. 2a, compared to control worms, 150 μg/ml JPYW-treated worms had a significantly increased mean lifespan during heat stress (5.82 ± 0.62 vs. 6.49 ± 0.81 h, *P* < 0.01). Thermotolerance was also elevated in aged worms. As shown in Fig. 2b, compared to the control treatment, JPYW treatment significantly increased the survival rate in aged worms (4.50 ± 1.20 vs. 5.29 ± 0.97 h, *P* < 0.01). Then, we determined whether JPYW also exerted protective effects on wild-type and aged worms under oxidative stress conditions. Interestingly, compared to the control treatment, JPYW treatment improved survival under mild to moderate oxidative stress but did not improve survival under severe oxidative stress. The results showed that JPYW-treated wild-type worms lived longer than control vehicle-treated worms under 0.6 to 0.8 mM hydrogen peroxide-induced oxidative stress (Fig. 2c). Significant differences were also observed between aged wild-type worms and aged control worms under 0.4 to 1.0 mM hydrogen peroxide-induced oxidative stress (Fig. 2d).

### Effects of JPYW on antioxidant enzyme activity

**Fig. 3:**
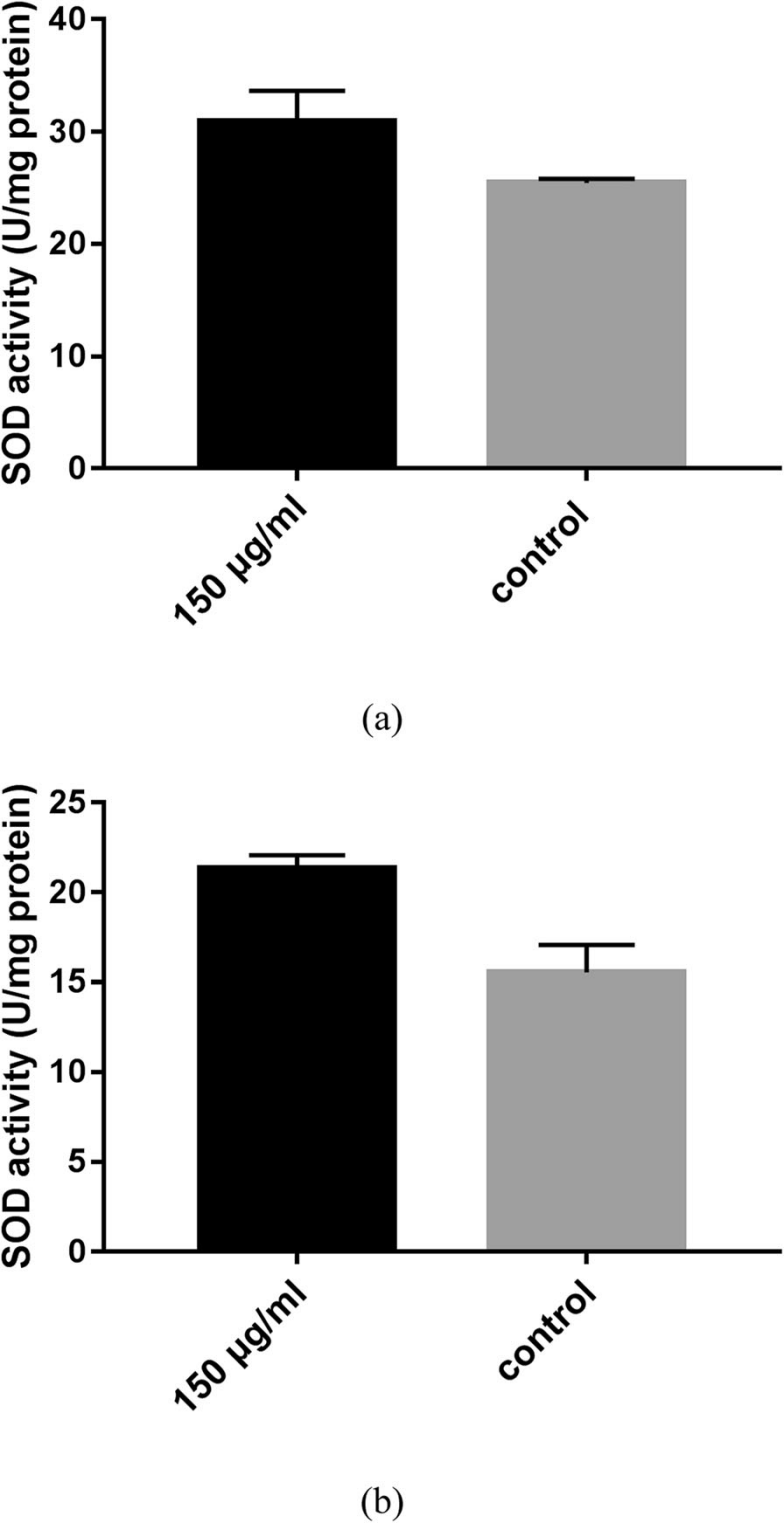
Effect of JPYW on SOD activity in *C. elegans* N2 worms. **a** SOD activity in *C. elegans* N2 worms. Quantitative comparisons showed that SOD levels were significantly higher in JPYW-pretreated worms than in control worms (25.44 ± 0.22 vs. 30.96 ± 1.53 U/mg of protein, *P* < 0.05). **b** SOD activity in aged *C. elegans* N2 worms. Quantitative comparisons showed that SOD levels were significantly higher in JPYW-pretreated aged worms than in control worms (15.54 ± 1.09 vs. 21.35 ± 0.52 U/mg of protein, *P* < 0.05)

To verify the possible mechanism by which JPYW mediated longevity extension and elevated stress tolerance, the activity of individual stress resistance proteins was investigated in wild-type worms and aged worms. In this study, we assessed the activity of antioxidant enzymes such as SOD. As shown in Fig. 3a and b, SOD was significantly upregulated in the presence of 150 μg/ml JPYW in both wild-type and aged worms compared to controls (*P* < 0.05).

### Effects of JPYW on aging-related factors

**Fig. 4:**
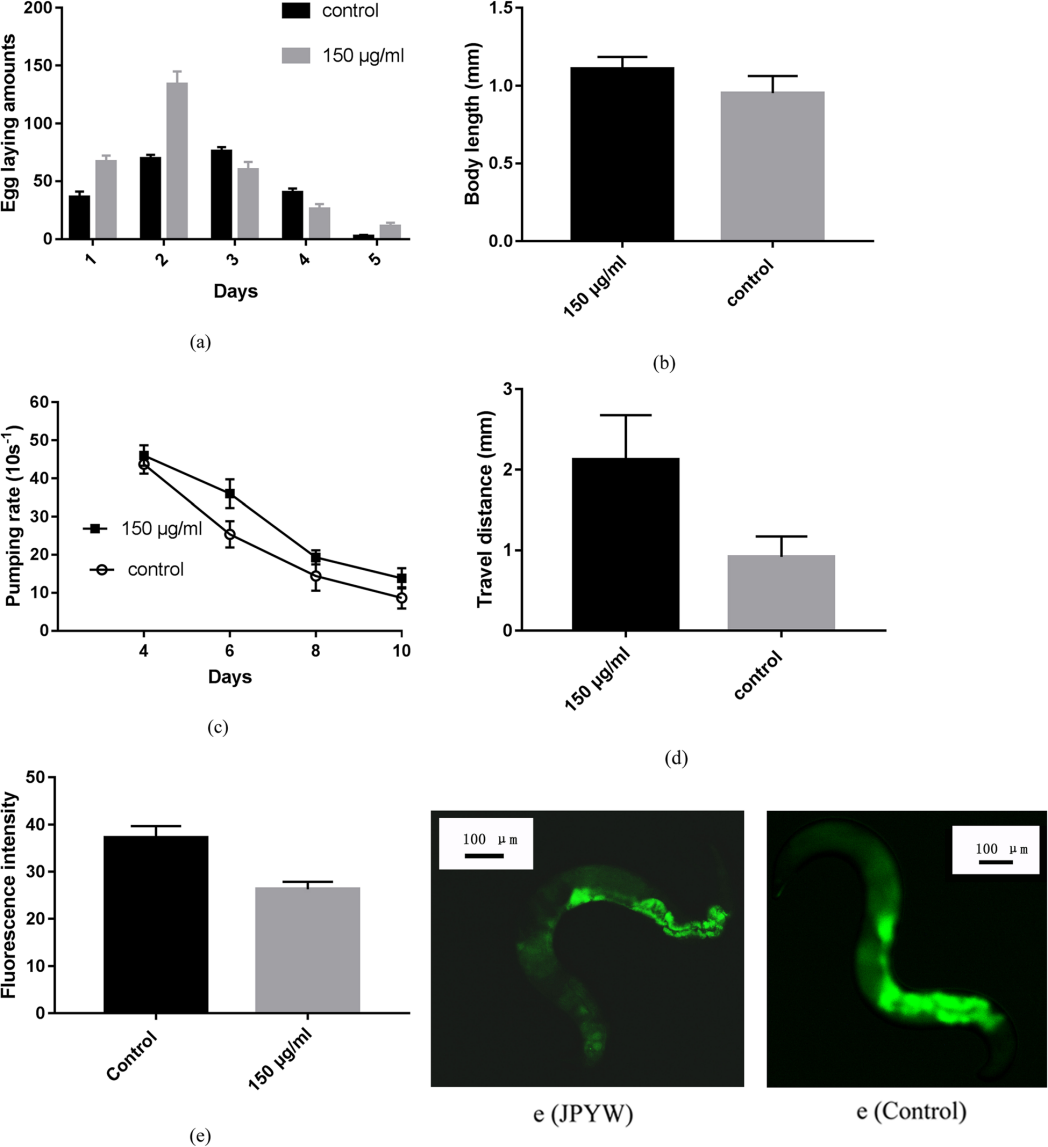
Effect of JPYW on aging-related factors. **a** Daily and total reproductive outputs. The progeny were counted at the L2 or L3 stage. JPYW treatment significantly increased the total progeny number (297.4 ± 15.3 vs. 223.8 ± 6.3, n = 5, *P <* 0.01) compared to the control treatment. **b** For the growth alteration assay, photographs were taken of the worms, and the body length of each animal was analyzed. A small but significant change in body length was detected after JPYW treatment compared to the control treatment (0.953 ± 0.035 vs. 1.108 ± 0.024 mm, *n* = 10, *P <* 0.05). **c** JPYW slowed the decline in pharyngeal pumping during aging. Worms were treated with 150 μg/ml JPYW and the pumping rates (pumps per 10 s) of 10 animals were scored in two trials (untreated vs. treated: day 6, *P* < 0.05; day 8, *P* < 0.05; day 10, *P* < 0.05; n = 10). **d** Body movement in wild-type N2 nematodes. Worm body movement was evaluated under a dissecting microscope for 20 s. The differences between the JPYW-treated worms and controls were significant (0.92 ± 0.08 vs. 2.13 ± 0.18 mm, n = 10, *P* < 0.01). **e** Fluorescence intensity of lipofuscin and autofluorescence on the 10th day of adulthood. Compared to that in control worms, the intestinal lipofuscin accumulation in JPYW-treated worms was reduced (37.29 ± 0.54 vs. 26.32 ± 0.35, *n* = 20, *P* < 0.01)

Previous study has indicated that lifespan was associated with reproduction, pharyngeal pumping, body size and motility in many species, such as *C. elegans* [27]. In this study, we found that JPYW treatment significantly increased the total number of progeny compared to the control treatment (297.4 ± 15.3 vs. 223.8 ± 6.3 progeny, *n* = 5, *P* < 0.01, Fig. 4a). In addition, a significant change in worm body length was detected after JPYW exposure (0.953 ± 0.035 vs. 1.108 ± 0.024 mm, *n* = 10, *P* < 0.05, Fig. 4b), suggesting that JPYW activity affects growth as well as fertility (Fig. 4a). Then, we assessed the muscle activity and the movement ability of the worms by recording the rate of pharyngeal pumping. The graph in Fig. 4c showed that the rate of pharyngeal contractions declined gradually with increasing age, and this aging-associated decline was attenuated by JPYW treatment compared to the control treatment (Fig. 4c). Then, we measured the body movements to estimate the healthspan of aged worms (worms that had been adults for more than 7 days) by recording the distances the worms traveled over 20 s. As shown in Fig. 4d, worm body movement was significantly higher in the JPYW group than in the untreated control group (0.92 ± 0.08 vs. 2.13 ± 0.18 mm, n = 10, *P* < 0.01), suggesting that the functional aging of worms is strongly delayed by JPYW. As shown in Fig. 4e, the fluorescence intensity of intestinal lipofuscin was significantly attenuated in the JPYW group compared to the control group (37.29 ± 0.54 vs. 26.32 ± 0.35, *n* = 20, *P* < 0.01).

### Effects of JPYW on aging-related gene expression

**Fig. 5:**
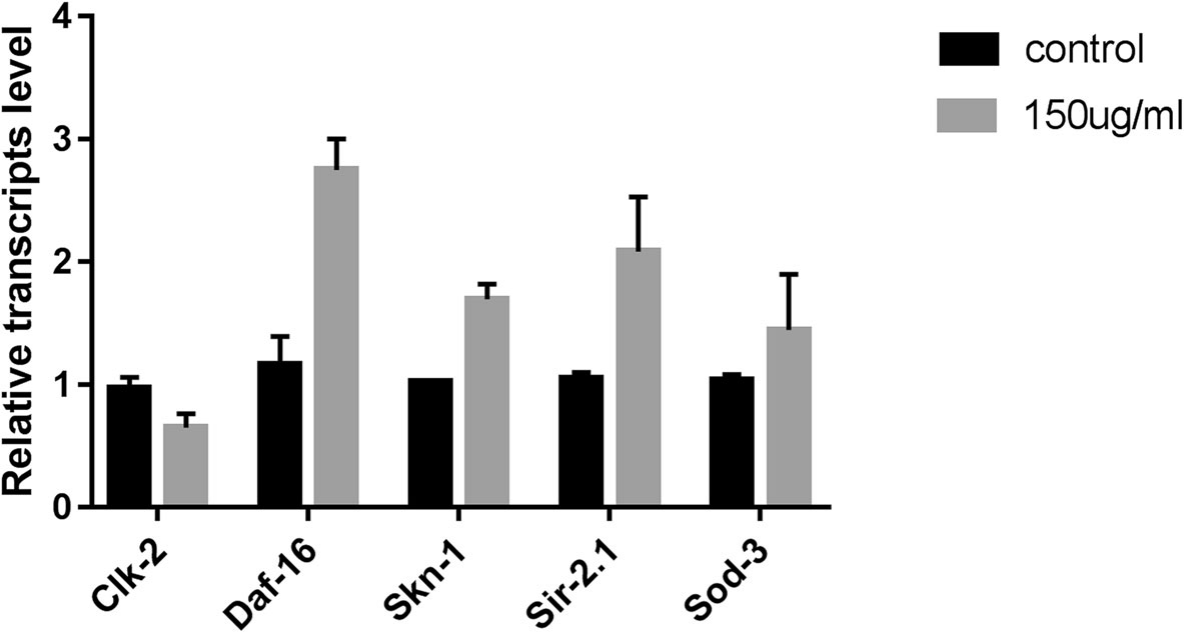
Effects of JPYW treatment on the expression of aging-related genes. The expression levels of aging-related genes were determined by qRT-PCR using the 2^−ΔΔCT^ method in worms with or without 150 μg/ml JPYW treatment at 20 *°C*. The graph shows the mean and SEM values from two independent experiments. Compared to the control treatment, JPYW treatment significantly changed the expression levels of the genes daf-16, clk-2, skn-1 and sir-2.1 (*P* < 0.05), but not those of the gene sod-3 (*P* > 0.05)

Pathways for the induction of stress-response genes that affect lifespan have been identified in *C. elegans*. JPYW treatment might improve survival by activating these genes. Treatment with JPYW can increase *C. elegans* lifespan through *sir-2.1*, which regulates this effect through *kat-1*-mediated fatty acid oxidation [28]. As shown in Fig. 5a, the expression level of the *sir-2.1* gene was significantly upregulated in JPYW-treated worms compared to control-treated worms. In *C. elegans*, two transcription factors, *daf-16* and *skn-1*, promote the expression of antioxidant or detoxification enzymes, enhance stress resistance and increase lifespan [29, 30]. JPYW treatment significantly increased the expression levels of the *daf-16* and *skn-1* genes compared to the control treatment, suggesting that JPYW may act in a manner that is dependent on these genes (Fig. 5a). JPYW treatment also significantly downregulated the expression level of *clk-2* compared to the control treatment, which may have slowed the shortening of telomere length in the JPYW-treated worms, resulting in increased lifespan. Surprisingly, compared to the vehicle control, JPYW significantly increased SOD activity, but it did not increase the expression of the *sod-3* gene.

## Discussion

In the present study, one control group (1% DMSO) and one experimental group (150 μg/ml) were used to explore the antiaging effects of JPYW and their underlying mechanisms in a *C. elegans* model. Since the experiments were not designed as noninferiority tests or superiority tests, a positive control group was not used. Each test in the study was performed at least two times to control for random effects and to ensure the repeatability and accuracy of the results. We found that JPYW treatment significantly prolonged the lifespan of wild-type worms under stress conditions. In addition, the lifespan of aged worms increased more significantly than that of wild-type worms under both normal and stress conditions. This result indicates that JPYW may have a strong antiaging effect and that JPYW therapy may be a useful antiaging treatment. As previously reported, most of the plants in JPYW have antiaging effects. For instance, *P. ginseng C. A. Mey,* one of the main herbs in this formula, has been proven to be very effective in delaying senility [31], and ginsenosides, the active ingredients in *P. ginseng*, have been proven to promote development and growth and to prolong lifespan of *C. elegans* [32]. In addition, ginsenoside Rg1, the main active pharmaceutical ingredient in *P. ginseng*, has been found to improve the antiaging ability of the hematopoietic microenvironment by enhancing the antioxidant and anti-inflammatory capacities of bone marrow stromal cells in a D-galactose-induced aged rat model and also to act on hematopoietic cells to protect them from aging [33, 34]. Pachymic acid, a main compound in *P. cocos*, can induce autophagy via the IGF-1 signaling pathway in aged cells to delay the aging process [35]. Additionally, nobiletin, an active ingredient in *Pericarpium Citri Reticulatae*, may ameliorate isoflurane-induced cognitive impairment and delay the aging process through antioxidant, anti-inflammatory and antiapoptotic effects via modulation of Akt, Bax, pCREB and BDNF in aging rats [36]. Finally, *C. cassia Presl* can increase *C. elegans* lifespan via insulin signaling and stress-response pathways [37], and the major chemical components of *C. cassia*, cinnamates, may promote adiponectin production during adipogenesis in human adipose tissue-derived mesenchymal stem cells and prevent skin aging [38]. JPYW may thus exert antiaging effects through the combined effects of all of its components.

Recently, antiaging medicine has aimed at not only simply increasing longevity but also extending healthspan. In this study, we showed that JPYW treatment effectively delayed aging-related declines in function, such as pharyngeal pumping, body movement, egg laying and development, compared with the control treatment, indicating that JPYW can enhance the healthspan of worms.

To explore the potential mechanisms by which JPYW exerts antiaging effects, SOD activity and aging-related gene expression were assessed in *C. elegans*. As was reported in the previous studies [39, 40], the oxidative stress caused by oxygen free radicals played an important role in aging, and eliminating free radical and enhancing oxidative stress resistance could delay senility. Our research indicated that compared to the control treatment, JPYW treatment elevated the activity of an antioxidant enzyme (SOD), which resulted in elimination of oxygen free radicals that might contribute to aging. Notably, previous studies have revealed that gene expression can change during aging in *C. elegans*. Using qRT-PCR, we confirmed that compared to control-treated worms, JPYW-treated worms exhibited upregulated expression of the antiaging genes *daf-16*, *skn-1*, and *sir-2.1* and downregulated expression of the proaging gene *clk-2*, while they did not exhibit changes in the antiaging gene sod-3. Overall, four key genes are involved in the ameliorative effects of JPYW on the aging pathway. The first, *daf-16* [41], is a part of *FOXO*-family transcriptional factor, which can regulate many target genes that can improve stress resistance and increase longevity. The second, *sir-2.1* [42, 43] belongs to *NAD*^*+*^-dependent histone deacetylases, which involves in regulating lifespan conservatively. As was previously reported, overexpression of *sir-2.1* can extend the longevity of *C. elegans* by suppressing the IIS pathway or activating *daf-16*. The third, *skn-1* [44], involves in regulating oxidative stress resistance and lifespan by encoding a worm homolog of *Nrf2*. The fourth key gene, *clk2* [45], reduces longevity and telomere length.

In the present study, JPYW upregulated the activity of the antioxidant enzyme SOD but did not significantly increase the expression of the relevant gene sod-3. This finding indicates that protein expression did not correlate with gene expression, which is an intriguing and unexplained phenomenon. The precise mechanisms underlying these results are uncertain, but it is known that some proteins are not encoded by only single genes. For example, SOD is encoded not only by the gene sod-3 but also by the genes sod-2, sod-1, etc. In addition, the process of gene regulation is complex and unclear. This issue requires further study, and this discrepancy is one of the limitations of our study. In addition, JPYW is a Chinese herbal compound that contains many complex components, such as steroid-like compounds, but no specific compound extracted from JPYW was tested in this study. Hence, it is not clear how many ingredients were related to the observed antiaging effects or how these active ingredients may have interacted. This uncertainty is another limitation of the present study. Further studies are warranted to identify the active ingredients in JPYW.

## Conclusions

In conclusion, this study demonstrated that JPYW, a TCM formula, increases stress resistance and promotes longevity in *C. elegans* by activating and repressing target genes related to aging, including *daf-16*, *sir-2.1*, *skn-1* and *clk-2*.

## Data Availability

The datasets generated and analysed during the current study are not publicly available since a follow-up study is undergoing, but are available from the corresponding author on reasonable request.

## Abbreviations

BCA: Bicinchoninic acid
*C. elegans*: *Caenorhabditis elegans*
cDNA: complementary DNA
CFDA: China food and drug administration
CGC: Caenorhabditis genetics center
DMSO: Dimethylsulfoxide
EDTA: Ethylenedinitrilotetraacetic acid
JPYW: Jianpi-yangwei
NGM: Nematode growth medium
qRT-PCR: quantitative real-time polymerase chain reaction
SEM: Standard error of the mean
SOD: Superoxide dismutase
TCM: Traditional Chinese medicine
Tris-HCl: Tris(hydroxymethyl)aminomethane hydrochloride
T-SOD: Total superoxide dismutase
